# Effects of human tissue acoustic properties, abdominal wall shape, and respiratory motion on ultrasound-mediated hyperthermia for targeted drug delivery to pancreatic tumors

**DOI:** 10.1080/02656736.2022.2091799

**Published:** 2022-07-19

**Authors:** Michael Gray, Laura Spiers, Constantin Coussios

**Affiliations:** a Institute of Biomedical Engineering, University of Oxford, Oxford, UK; b NIHR Oxford Biomedical Research Centre, Oxford, UK; c Department of Oncology, University of Oxford, Oxford, UK

**Keywords:** Ultrasound, hyperthermia, pancreas, treatment planning, simulation

## Abstract

**Background:**

PanDox is a Phase-1 trial of chemotherapeutic drug delivery to pancreatic tumors using ultrasound-mediated hyperthermia to release doxorubicin from thermally sensitive liposomes. This report describes trial-related hyperthermia simulations featuring: (i) new ultrasonic properties of human pancreatic tissues, (ii) abdomen deflections imposed by a water balloon, and (iii) respiration-driven organ motion.

**Methods:**

Pancreas heating simulations were carried out using three patient body models. Pancreas acoustic properties were varied between values found in the literature and those determined from our human tissue study. Acoustic beam distortion was assessed with and without balloon-induced abdomen deformation. Target heating was assessed for static, normal respiratory, and jet-ventilation-controlled pancreas motion.

**Results:**

Human pancreatic tumor attenuation is 63% of the literature values, so that pancreas treatments require commensurately higher input intensity to achieve adequate hyperthermia. Abdominal wall deformation decreased the peak field pressure by as much as 3.5 dB and refracted the focal spot by as much as 4.5 mm. These effects were thermally counteracted by sidelobe power deposition, so the net impact on achieving mild hyperthermia was small. Respiratory motion during moving beam hyperthermia produced localized regions overheated by more than 8.0 °C above the 4.0 °C volumetric goal. The use of jet ventilation reduced this excess to 0.7 °C and yielded temperature field uniformity that was nearly identical to having no respiratory motion.

**Conclusion:**

Realistic modeling of the ultrasonic propagation environment is critical to achieving adequate mild hyperthermia without the use of real time thermometry for targeted drug delivery in pancreatic cancer patients.

## 1. Introduction

There has been little improvement in outcomes for patients with pancreatic ductal adenocarcinoma (PDAC) over the last 40 years [1]. Pharmacological advances such as checkpoint inhibitors have been used with success in other areas of oncology. However, the tumor microenvironment of pancreatic cancers remains a barrier to drug delivery. Abundant desmoplastic stroma raises interstitial pressure to cause vascular collapse and limit tumor perfusion. Even if drugs can penetrate, pancreatic stellate cells (PSC) of the stroma help sustain an immunosuppressive environment.

An increasingly popular approach to address limitations of PDAC cytotoxic therapy is to employ localized heating [2–6], which can induce several anti-cancer effects. The immune response from mild thermal injury can help combat the ‘cold’ immune environment. Localized heating causes vasodilation to improve tumor blood flow for enhanced drug delivery, and hyperthermia can also be combined with thermosensitive drugs for targeted release of therapeutic payload [7,8].

Multiple methods of inducing hyperthermia in pancreatic tumors have recently been evaluated in clinical trials, including radiowave [9], microwave [10], and focused ultrasound approaches guided both by MRI [11] and diagnostic ultrasound [12]. This also includes the current PanDox trial [13], a Phase I trial investigating whether Focused Ultrasound (FUS)-induced mild hyperthermia (>39.5 °C) enhances delivery of doxorubicin encapsulated within thermosensitive liposomes (ThermoDox^®^, Celsion Corporation, USA) to non-resectable PDACs. FUS-mediated mild hyperthermia typically involves generation of spatial peak temporal average intensities (I_spta_) between 50 and 500 W/cm^2^, resulting in transient temperature elevations of 4–5 °C [14]. FUS is relatively quick, inexpensive, and less invasive than other commonly used modalities for hyperthermia induction.

There has been considerable recent work using MR guided systems to deliver targeted hyperthermia with anatomically registered monitoring of temperature elevation [15,16]. The application of MR-guided FUS systems is limited primarily by cost, and to a lesser extent by technical issues with motion and monitoring in adipose tissue [17,18]. Ultrasound-guided systems may also be used, but unlike an ablation scenario where hyperechoic tissue confirms the desired effect, there is no clinically reliable way to use ultrasound imaging to indicate the modest temperature elevations of interest in mild hyperthermia. The alternatives are to use invasive thermometry, or to use predictive models to define treatment parameters. The former was used to validate the latter for treatment of liver tumors as part of the TARDOX trial [19]. This was a first-in-human study for the safety and feasibility of drug release from thermosensitive liposomes (ThermoDox™) to tumors in the liver and utilized focused ultrasound coupled to a B-mode US system for simultaneous treatment and guidance. Treatment plans were constructed using inputs of anatomical data from CT and MRI, along with acoustic and thermal properties for the constituent tissues. With these inputs, the model was used to predict acoustic pressure and temperature maps of the target area, and to generate personalized ultrasound treatment plans (power, duty cycle, and therapeutic treatment volume) for enhanced drug delivery to a tumor target [20]. A key finding of the TARDOX trial was that patients treated using a modeling-only approach experienced comparable enhancements in drug delivery to those treated with invasive thermometry.

The PanDox trial requires a modified version of the validated TARDOX treatment planning model to define FUS exposure parameters for hyperthermia in the pancreas. As the treatment concept is ultrasound guided and thermometry-free, there are several additional aspects to consider.

Firstly, ultrasound treatments such as liver ablation have been performed for several years, and acoustic properties of the liver to support planning models are readily available [21,22]. Although thermal properties of the pancreas are known from human postmortem and *ex vivo* porcine organs, data for acoustic properties of the pancreas are more limited [23–25]. Published values for sound speed are based on measurements taken from healthy porcine specimens, performed at 23–26 °C [26]. Similarly, the limited studies into acoustic attenuation have been performed in bovine organs at 24 °C [27]. It is not known whether these accurately reflect properties of normal or pathological human pancreatic tissues.

Accurate FUS application to abdominal organs is challenging due to the presence of the gas-filled gastro-intestinal tract in the acoustic pathway and respiratory movement of the intended target out of the beam path [28]. In the PanDox trial participants will lie prone, compared to the lateral/supine positions used in TARDOX. To clear the acoustic path for accurate targeting and minimization of organ damage, a degassed water balloon may be deployed against the abdominal wall to gently move the bowel and stomach [29]. The resulting curvature of the fat and muscle layers may affect the beam focus location and size, and is therefore an additional consideration in the updated model described here.

Finally, unlike the liver, the pancreas is a retroperitoneal organ and was previously thought to be modestly affected by respiratory movement. However, radiological assessment with four-dimensional CT has demonstrated cranio-caudal displacement of the pancreatic head by up to 7.6 mm ± 3 mm in each direction [30]. In patients with chronic pancreatitis, similar movement measured using fluoroscopy has been has high as 14.4 mm ± 9.1 mm [31]. Although pancreatic head movement was reduced when patients lay prone (as will be the preferred position for PanDox) [32], this movement change is still greater than the diameter of the ultrasound focus. This could lead to over-heating the target in some locations and under-heating in others and may increase the risk of off-target heating effects.

Various approaches to minimize organ movement have been studied and are summarized elsewhere [33]. Our trials of FUS-mediated hyperthermia have used high-frequency jet ventilation (HFJV) to control patient breathing for minimization of organ motion. Using this technique, small tidal volumes are delivered at high frequency to ‘jet’ gases into the lungs for rapid exchange but with little volume change, minimizing diaphragm movement and subsequent organ displacement. Modeling the effect of respiratory motion on heating the pancreas target is of clinical importance as jet ventilation requires general anesthetic, and therefore if it can be shown that this is not required, this would result in less invasive treatment for patients and elimination of associated anesthetic risks.

As part of the effort to deliver targeted hyperthermia to pancreatic tumors in a safe and effective manner for the PanDox trial, this work presents simulation studies that introduce three new aspects to the previously validated hyperthermia model: (1) newly measured human pancreas acoustic properties derived from freshly excised PDAC tumors and healthy tissue, (2) abdominal wall curvature alterations induced by a water balloon and (3) respiratory-induced target motion during moving-beam FUS treatment.

## 2. Methods

This section begins with the PanDox treatment concept for targeted drug release in pancreatic tumors, including a description of the clinical FUS system and how treatment is to be administered. The treatment planning model is then presented, followed by discussions of the three new technical issues in this study: newly acquired human pancreas acoustic properties, abdomen tissue curvature, and respiratory motion.

### 2.1. Pandox treatment concept

**Figure 1.**
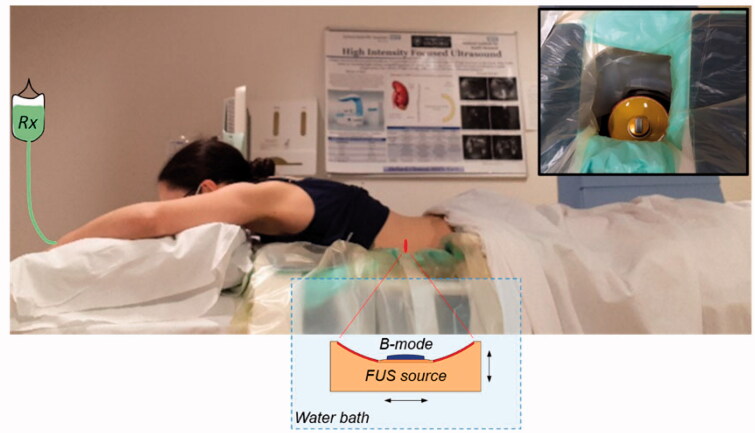
Illustration of the PanDox treatment concept. The patient lies prone over the JC200 water bath containing a FUS source fitted with a coaxial B-mode probe (inset photo). For induction of mild hyperthermia, the FUS source is continuously scanned under B-mode guidance to cover the prescribed treatment volume.

The treatment is conducted with the patient lying prone on the bed of an ultrasound-guided system (model JC-200, Haifu Technology Company, Chongqing, China) which employs a fixed focus high power source (‘FUS source’, 200-mm diameter, 0.96 MHz) with a coaxially configured curvilinear imaging transducer (‘B-mode’). The transducers sit below the treatment bed within a degassed and temperature-controlled water bath that facilitates coupling of the ultrasound fields to the patient (Figure 1). A degassed water balloon within the bath may be deployed to optimize the beam path (see Section 2.4). Once the target has been identified using B-mode imaging, minimization of respiratory-induced motion of the target volume and surrounding tissues is achieved using administration of general anesthesia under HFJV. Final mapping of the treatment volume is then completed, again under B-mode guidance.

FUS is first applied to pre-warm the target region for 5–20 min, depending on the size of the tumor and its margins. ThermoDox™ is then infused intravenously over a 30-min period whilst FUS continues. The target volume is repeatedly treated with FUS for up to 120 min from the start of infusion in order to release as much drug as possible in the tumor whilst the circulating ThermoDox™ concentration is sufficiently high. This two-hour time scale is based on prior clinical confirmation of Thermodox™ pharmacokinetics [19].

### 2.2. Treatment planning model

**Figure 2.**
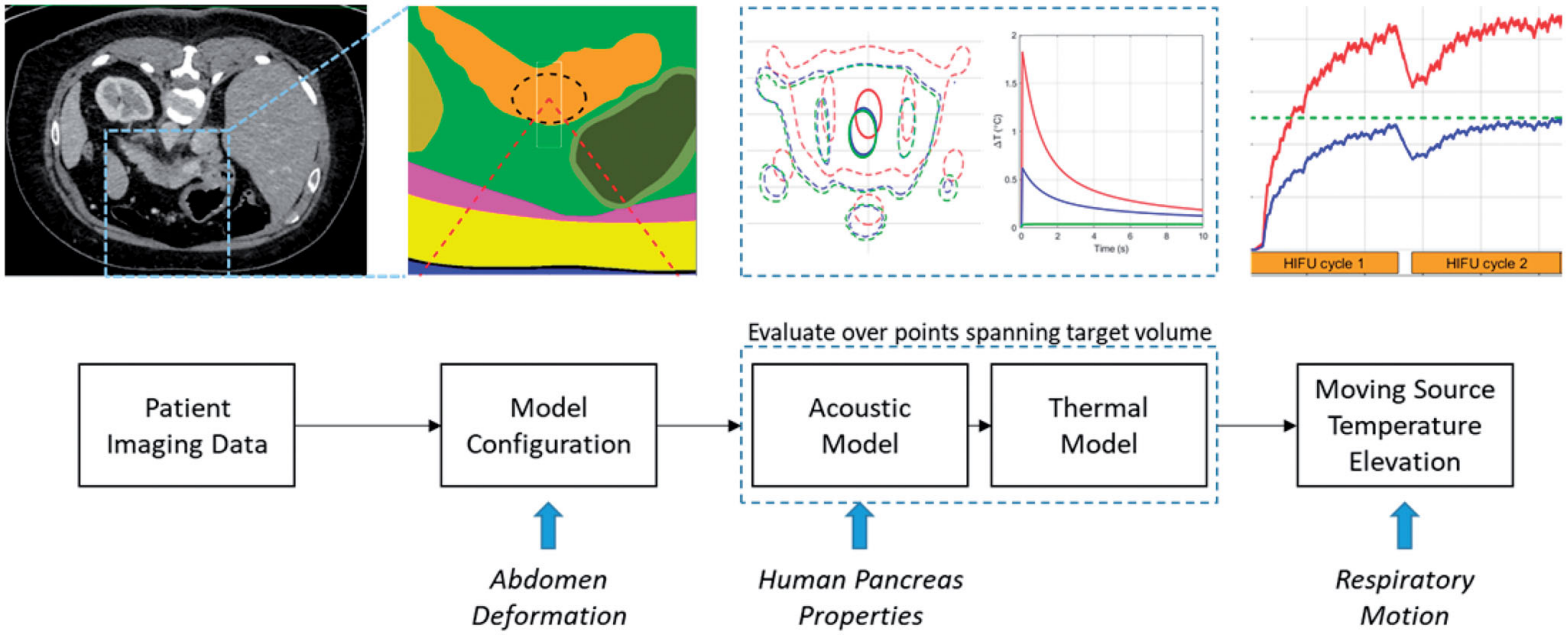
Illustration of the treatment planning model process. Patient CT data are segmented and used to define the geometries for 3 D finite element models. Pressure fields are used to calculate heat generation terms which are subsequently applied to thermal finite element models. Their four-dimensional outputs (temperature history in three dimensions) are used to simulate volumetric hyperthermia by moving the thermal data according to a prescribed trajectory and rate. New features in the present study are shown at the bottom of the figure (italic text) and are aligned with the part of the model where they are introduced.

A computational planning model was developed for the TARDOX study to estimate pressure and temperature fields, and to specify FUS parameters for ultrasound-mediated mild hyperthermia that would enable efficient release from thermally sensitive liposomes (>39.5 C). The acoustic and thermal components of the modeling scheme were validated with *in-vitro* experiments, and the model-based treatment planning approach was validated through comparison with clinical thermometry results [19]. Details of the modeling approach and its validation are described in [20] and are summarized here (Figure 2). The process involves computations performed in MATLAB (Mathworks, Natick, MA, USA) and finite element models run in PZFlex (v2015, OnScale, Redwood City, CA).

#### 2.2.1. Acoustic models

The simulation process begins with importation of patient contrast CT image data into MATLAB and segmentation into constituent tissues using in-house code. Material categories and their assigned values are listed in Table 1, which includes data for healthy and pathological human pancreas samples collected for PanDox as discussed in Section 2.3.

**Table 1.** Acoustic and thermal properties used for pancreas treatment simulations.

Tissue	Density (kg/m^3^)	Sound Speed (m/s)	Attenuation (dB/cm)	Thermal Conductivity (W/m/^^^C)	Specific Heat (J/kg/^^^C)	Perfusion (kg/s/m^3^)
Pancreas						
Literature, Normal	1087 [34]	1591 [26]	0.79f^1.12^ [27, 35]	0.458 [23, 36]	3188 [23]	14.9 [37–41]
Literature, Tumor	1087 [34]	1591 [26]	0.79f^1.12^ [27, 35]	0.478 [36]	3188 [23]	5.2 [37–41]
Human Data, Normal	–	1549 [42]	0.58f^1.51^ [42]	–	–	–
Human Data, PDAC	–	1555 [42]	0.50f^1.59^ [42]	–	–	–
Duodenum wall	1045 [43]	1535 [34]	0.43f [43]	0.53 [43]	3698 [43]	16.67 [43]
Stomach wall	1045 [43]	1535 [34]	0.43f [43]	0.53 [43]	3698 [43]	6.75 [43]
Liver	1079 [34]	1586 [34]	0.60f [44–50]	0.57 [51]	3800 [51]	10.7 [51]
Spleen	1089 [34]	1567 [52,53]	0.52f [54]	0.53 [34]	3596 [34]	30.8 [55]
Abdominal muscle	1050 [56]	1547 [56]	0.59f^1.53^ [57–59]	0.55 [51]	3524 [51]	0.48 [51]
Abdominal fat	950 [56]	1478 [56]	0.59f^1.53^ [57–59]	0.19 [51]	2353 [51]	0.56 [51]
Connective tissue	1027 [34]	1545 [60]	1.26f [34]	0.39 [34]	2372 [34]	0.0
Skin	1120 [56]	1613 [56]	1.57f [56]	0.34 [61]	3391 [34]	0.0

The first acoustic calculation step is the generation of the radiated pre-focal field produced by the FUS source in open water (free field) when driven at its fundamental frequency (0.96 MHz). The calculation is run using an axisymmetric geometry matching the dimensions of the FUS source (200 mm outer radius, 80 mm inner radius, 170 mm radius of curvature). The pre-focal field is used to define a boundary load for the three-dimensional patient body models that followed. Separation of the drive from the body model allows for improved control over model size and run time. Body models are uniformly meshed in all directions with a grid dimension of 95 µm based on preliminary convergence studies.

3-D pressure fields in the body models are exported to MATLAB for calculation of heat generation terms from:

(1) $$\dot{q}=2\alpha I_{pa}$$

(2) $$I_{pa}=\frac{1}{\rho c\tau_{p}}\int_{t=0}^{\tau }p^{2}dt$$

where the intensity $I_{pa}$ is calculated with pulse time *τ*_*p*_ and total record time *τ*.

All acoustic models are run with a linear formulation, and in combination with Equation 2, the nonlinear contributions to heating are ignored. This set of assumptions had been previously found to cause heat generation and temperature elevation errors <10% for treatments employing the parameters of interest in this study (peak negative pressure <8 MPa, 0.96 MHz fundamental frequency) [20]. Absorption and attenuation are taken to be equal under the assumption that scattering contributions to total attenuation are locally absorbed [43].

#### 2.2.2. Thermal models

The heat source terms found in the previous step are imported back into a PZFlex bioheat transfer finite element calculation incorporating thermal tissue properties shown in Table 1. Temperature elevations were found by driving each heat source for 20 ms (1/5th the typical pulse repetition period employed in the TARDOX trial) and calculating the temperature field for 240 s. Infinite thermal elements were applied at the model boundaries, the starting field was at a uniform temperature, and all thermal properties were taken to be independent of temperature. A uniform thermal grid size of 120 µm was chosen, corresponding to the distance traveled by the FUS source in 20 ms (the shortest pulse length employed with the JC200) when traveling at the maximum available translation speed of 6 mm/s.

**Figure 3.**
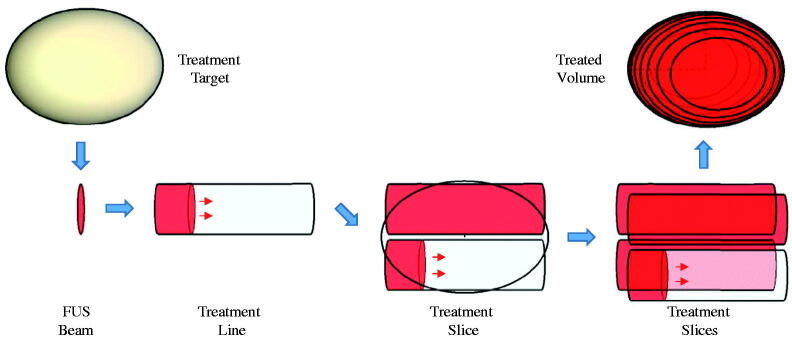
Illustration of subdivision of a target volume by lines and slices to cumulatively achieve hyperthermia with a moving FUS source beam.

Temperature fields generated in PZFlex for individual heat source distributions (corresponding to a single FUS source position with respect to the fixed patient body) were superposed in MATLAB to form volumetric temperature elevation estimates (Figure 3). Specifically, a line scan temperature profile was synthesized by adding fields from successive source positions, with sequential time delays defined by the ratio of thermal grid step size and the source translation speed. Two-dimensional slices were synthesized from the addition of line scans at multiple tissue depths, and volume scans were formed from the addition of multiple slices. The validity of using superposition was verified for a scan line by comparison with an explicit translating source model generated entirely within PZFlex [20].

A ‘cycle’ of hyperthermia treatment corresponds to the full coverage of the target volume with the moving beam. These cycles are repeated for up to 1.5 h without modification unless needed based on feedback from the B-Mode system (e.g., if the patient’s position has shifted). For this study, two cycles were run with a 30 s separation time – the first as the initial preheating stage, and the second as a typical maintenance cycle.

The ultimate output of the model is a temperature history in a three-dimensional grid encompassing the target tumor, along with statistical descriptions of volume heating and cumulative exposure. The administrative burden associated with transferring data between programs as described above is more than offset by the enhanced control over problem size and memory allocation. All processing described here is conducted on a single PC workstation with 256 GB RAM. While the calculations are tailored to run on this system, the broader objective carried over from TARDOX was to make trustworthy pressure and temperature estimates in support of hyperthermia treatment planning in a timeframe within the window between patient screening and scheduled treatment (as few as two weeks).

Treatment-cumulative metrics were computed from the compiled temperature histories described above, including: *T*_50_, *T*_10_ and *T*_90_ (median temperature, as well as temperature exceeded in 10 and 90% of the treatment volume, respectively), *T*_max_ (maximum temperature), and cumulative thermal dose (CEM43, [62]).

#### 2.2.3. Patient specific models

The above techniques were applied to three different patient models chosen for the relative clarity with which the pancreas could be identified in the image sets and for their differences in the types of tissue between the abdominal wall and the pancreas body. Anonymized CT scans for each patient were acquired with consent from the PanDox and TARDOX trials and processed as in Section 2.2. To help limit the otherwise broad parameter range in this study, a standard target was used for all patient models. The target was an elliptical volume with major axis of 28 mm and minor axes of 10 mm. The standard target was placed mid-body in each pancreas model and given the properties of PDAC tissue. Illustrations of the segmented models are presented in Section 3.

### 2.3. Human pancreas acoustic properties

Normal and pathological human pancreatic issue samples were made available post-surgery in cooperation with a hospital pathology laboratory after patient consent under an approved protocol (Oxford Center for Histopathology Research 19/A100). Propagation through samples of various thicknesses and pathologies was observed using a set of custom-built calipers based on an existing design concept [63]. Samples were held in a water bath at physiologic temperature, and the acquired through-transmission data were processed to estimate sound speed and attenuation. Summary results are included in Table 1, with details of the methods and outcomes presented elsewhere [42].

Using one patient model (M1), the full treatment planning process was evaluated using sound speed and attenuation from the human data set and from the literature. For these simulations, all other properties (e.g., thermal) were taken from the literature. *In situ* distributions of acoustic pressure and moving beam volumetric heating were calculated and compared specifically to observe the potential errors encountered by assuming the literature values were correct.

### 2.4. Balloon deformation

**Figure 4.**
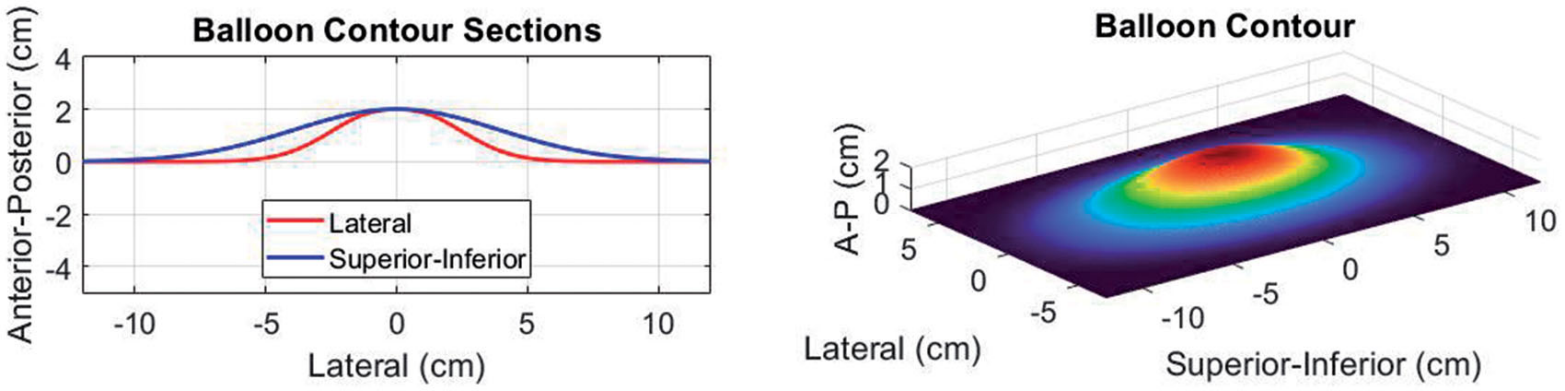
Illustration of the water balloon displacement profile applied to the patient models.

To simulate the effect of water balloon induced displacements on the abdominal wall, the three-dimensional wall profile for each body model was deformed using the shape function shown in Figure 4. This shape was defined based on the actual size of the balloon (10 × 17 cm uninflated) and the deformed shape (2 cm typical peak deflection) in the B-mode imaging plane as observed with two different cases during a separate trial. The observed shape in the superior-inferior (S-I) direction (along the JC200 imaging probe aperture) was fit with a Gaussian profile:

(3) $$g(x)=\exp(-0.5\;{\left(x\;(N-1)/(2\;\mathrm{alpha})\right)}^{2})$$

where x is a vector of length (N) describing the centered grid of locations where the shape is defined, and alpha (the width factor) was set to 3.45. In the lateral direction, a second Gaussian was used with alpha = 5.37 to conform to the elevational shape of the imaging probe and gradually taper to zero thereafter.

The deformations were applied to the abdominal wall without altering tissue layer thicknesses, and the centerline distance from skin to target was not changed. This latter approach was intended to simplify comparisons with baseline abdomen morphologies and is consistent with the observation in limited patient screenings that the skin to target distance may not be substantially different when the balloon is installed. In clinical practice the balloon is intended to collapse the stomach and push gas-bearing organs out of the FUS cone, but in the model study, no gases were included. The primary question was whether the tissue curvature and resulting refraction would impact the characteristics of the transmitted acoustic field.

### 2.5. Respiratory motion

**Figure 5.**
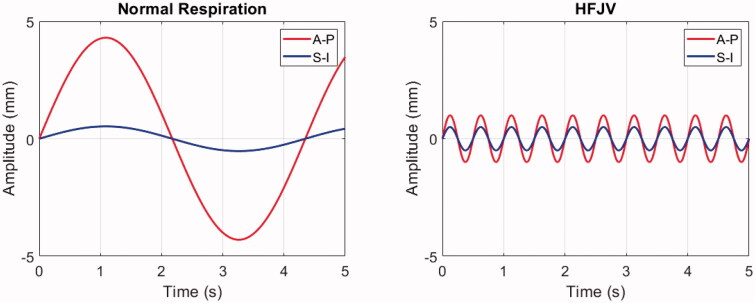
Respiratory-induced pancreas displacement profiles for normal respiration (left) and HFJV (right) in a 5.0 s interval. Anterior-posterior (A-P) and Superior-Inferior (S-I) components are shown in red and blue, respectively. No lateral displacements were included in the respiratory motion simulations.

The impact of respiratory motion on delivery of mild hyperthermia was assessed by shifting the time-dependent position of the ultrasound beam in the pancreas according to two pre-defined profiles (Figure 5). The first was for normal respiration, with values for a patient in the prone position (8.6 mm S-I, 1.1 mm A-P) [32] and an assumption of a 0.23 Hz breathing frequency [64]. The second was for HFJV, using a frequency of 2.0 Hz [65] and displacements of 2.0 mm (S-I) and 1.0 mm (A-P) [66]. In both cases, the displacements were taken to be sinusoidal [67] with respect to time (t):

(4) $$d_{SI}(t)=A_{SI}\text{sin}\left(2\pi ft\right),\;d_{AP}(t)=A_{AP}\;\text{sin}(2\pi ft)$$

where $A_{SI}$ and $A_{AP}$ are the displacement amplitudes in the superior-inferior and anterior-posterior directions, respectively, and f is the breathing frequency. In addition, left-right (lateral) motion was neglected, no shape or volume changes in the moving media were applied, and no adjustments were made for refraction or attenuation changes that could occur because the tissue was shifting with respect to the FUS field. Heating statistics were calculated as before and compared with heating in the absence of motion.

## 3. Results

### 3.1. Pancreas acoustic properties

Changes in the acoustic properties of targeted tissues may impact the in-situ pressure, intensity, and energy deposition rate. Critically, the ability to predict temperature elevations hinges on the latter, while initiation of cavitation and generation of radiation forces relate to the former. The acoustic properties for pancreas and PDAC tumor tissues listed in Table 1 show that in comparison with existing mammalian tissue data in the literature, the *ex-vivo* human data from our companion study has modestly lower sound speeds (<3%) and approximately 1/3rd lower attenuation coefficients: (0.79 vs. 0.50 dB/cm at 1 MHz = 0.091 vs. 0.058 np/cm at 1 MHz). The use of the human sound speed value may modify the location of the transmitted beam *via* refraction and cause small decreases in transmitted pressure. However, the dominant effects are expected to come from lower human data attenuation, both in the increased transmitted pressure and intensity and the lower energy deposition rate when taking the attenuation and absorption to be equal.

**Figure 6.**
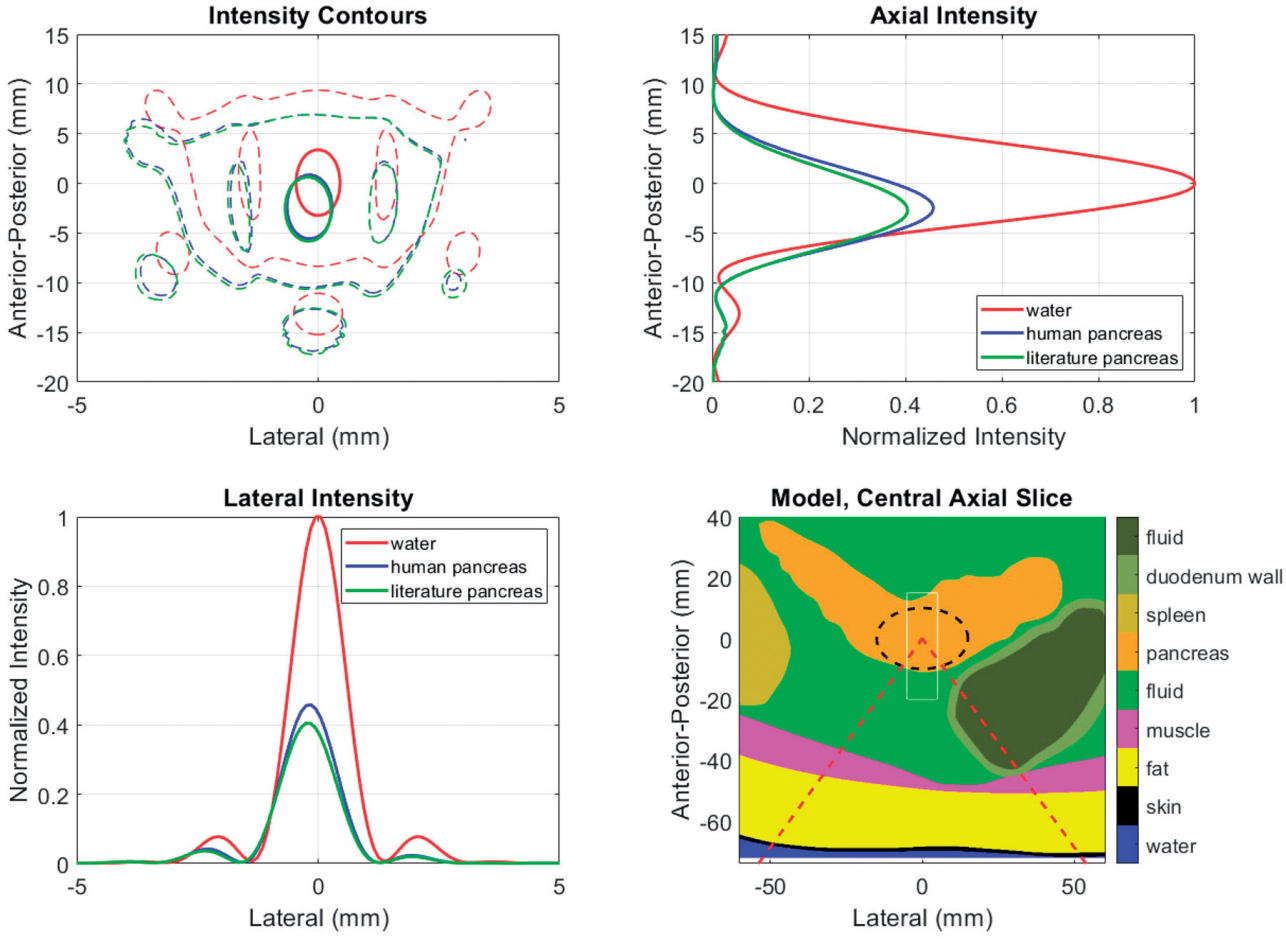
Acoustic intensities for patient model M1 using literature and newly acquired human pancreas acoustic properties. The model geometry (lower right) shows the distribution of tissues and includes the ‘cone’ of the incident HIFU beam (dashed red), the PDAC target (dashed black) and the window in which intensities are displayed in the other panels (thin white). The intensities are shown in the central axial plane of the model (upper left) at levels of 0.7 (thick solid) and 0.033 (thin dashed) relative to the maximum. Axial (upper right) and lateral (lower left) intensity profiles are shown at the level of each model’s maximum value.

Figure 6 shows the acoustic intensity patterns in patient model M1, which has the deepest tumor target. The contours in the central axial plane of the model show that propagation through the tissues causes a similar degree of refraction (2.4, 2.8 mm for human and literature values, respectively), but the beam pattern features are minimally impacted relative to that of an ideal result in water. The axial and lateral intensity profiles indicate that the literature values yield approximately 12% lower focal intensity than the human data, as expected.

**Figure 7.**
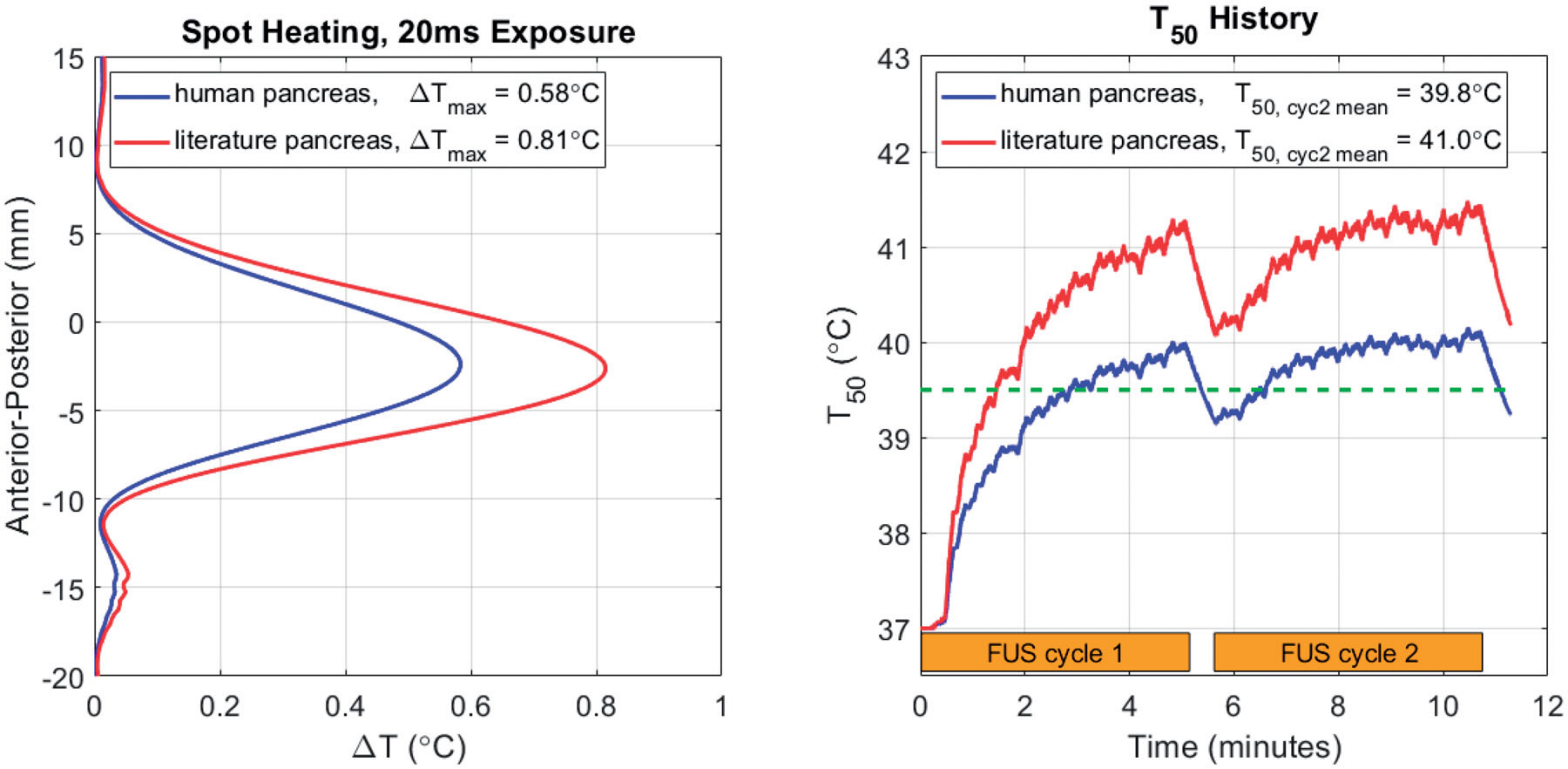
Temperature elevation simulation results with patient model M1 using literature and newly acquired human pancreas acoustic properties. Left: Anterior-posterior (depth) temperature distribution in the lateral center of the target after 20 ms of exposure, with the peak values listed in the legend. Right: T_50_ histories over two treatment cycles indicated by orange bars. Cycle-2 mean *T*_50_ values are listed in the legend. The dashed green line indicates the threshold temperature for drug release from ThermoDox™.

Temperature elevations for stationary and moving beam exposures in model M1 are shown in Figure 7. The HIFU input power was chosen under the assumption that the literature properties were correct, and with the goal of reaching a median temperature elevation of 41.0 °C in the target volume. The treatment consists of two identical cycles separated by 30 s, with the first serving to ‘preheat’ the target and subsequent cycles serving to maintain the desired temperature range.

As seen in the left panel of Figure 7, after 20 ms of HIFU exposure, the peak temperature elevation with the human properties is 72% of the peak obtained with the literature values as expected from the relative intensities and absorption coefficients. When the treatment volume is heated with the moving beam trajectory described in Section 2.2.2, the median temperature during the second cycle of treatment reaches the prescribed target value of 41.0 °C when literature pancreas values are assumed (Figure 7, right). However, when the human pancreas properties are used, the cycle-two time-averaged T_50_ value is 39.8 °C, which is just above the 39.5 °C release threshold for ThermoDox™. *T*_90_ values were 39.3 and 38.6 °C for literature and human pancreas simulations, respectively. In other words, assuming that the literature values were correct could result in conditions that were only borderline sufficient for drug release throughout the target tumor.

### 3.2. Abdomen deformation

**Figure 8.**
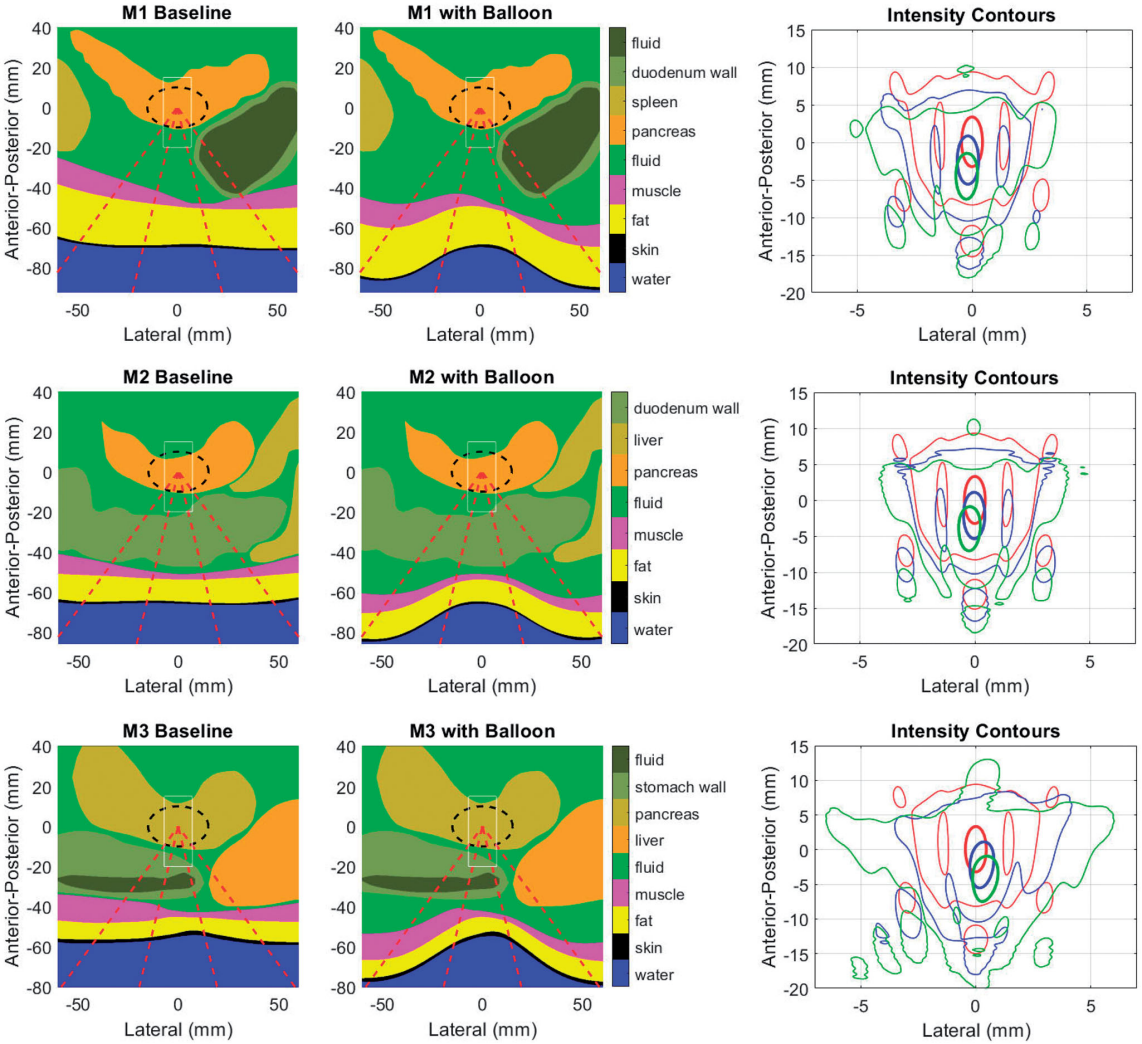
Geometries for the baseline (left) and balloon-deformed (center) configurations of each patient model. Overlaid lines indicated the annular ‘cone’ of the incident HIFU beam (dashed red), the PDAC target (dashed black) and the window in which intensities are displayed in the right-side panels (thin white). Intensity contours at 0.7 (thick line) and 0.033 (thin line) are overlaid in the same central axial plane of the models as displayed in the left and center panels, with colors denoting an idealized water path (red), baseline tissue (blue) and balloon-deformed tissue (green). Upper row: M1 (70 mm target depth), Middle row: M2 (66 mm target depth), Lower row: M3 (55 mm target depth).

Illustrations of the baseline (as observed in original CT scans) and balloon-deformed geometries are shown in Figure 8 for the three patient models employed in this study. The rightmost panel for each model shows the intensity contours in the central axial plane of each model, corresponding to the geometries shown left and center, and a set of accompanying field statistics is compiled in Table 2. In these examples, the beam was targeted to the middle of the PDAC target indicated by the dashed black line in the left and center column images.

**Table 2.** Acoustic field simulation statistics for each patient model.

Model	Target depth (mm)	Depth shift (mm)	p_rms_ re Water (dB)	Shape loss (dB)	ML FWHM (%)	Peak sidelobe (dB)	P_SL_/P_TOT_ (%)
M1							
Baseline	70	−2.4	−3.4	0.5	4	−10.4	45
Balloon	70	−4.5	−4.5	1.2	4	−8.5	50
M2							
Baseline	66	−2.3	−2.6	0.4	4	−11.3	46
Balloon	66	−4.6	−3.6	1.3	8	−9.4	54
M3							
Baseline	55	−2.3	−3.8	1.6	12	−9.1	46
Balloon	55	−4.2	−6.1	3.5	28	−5.1	71

Depth Shift: change in depth of p_rms_ relative to water; negative numbers are toward the HIFU source.

p_rms_: spatial peak of the root-mean-square pressure normalized to that of water.

Shape Loss: contribution to total reduction in p_rms_ due only to tissue refraction.

ML FWHM: Change in main lobe full width half maximum in the lateral plane relative to water. Positive values indicate expansion.

P_SL_/P_TOT_: ratio of sidelobe and total powers calculated at depth of spatial peak p_rms_.

Although the specifics vary, the major trends in all three patient models consist of proximal shifting of the main lobe (toward the FUS source) and increased power deposited in the sidelobes, with both effects amplified when the balloon deformations to the abdomen are applied. The extent of proximal shifting of the main lobe peak relative to that which observed in a lossless water environment was between 2.3 and 2.4 mm when using the baseline abdomen geometry. These values grew to between 4.2 and 4.6 mm with the balloon, which is approximately half the −6 dB length of the pressure main lobe in water. As seen in the intensity contours, a smaller degree of field peak shifting was observed in the lateral plane, all of it being less than 0.5 mm.

The peak pressure losses, quantified by the spatial peak of the root mean square pressure normalized to the value in water, vary from 2.6 to 3.8 dB for the baseline tissue. These values grow to as much as 6.1 dB for model M3 when the balloon deformations are applied. To understand the relative contributions to the total focal loss from attenuation and refraction-induced decorrelation, an additional set of models was run without tissue attenuation. The ‘shape loss’ metric in Table 2, which is the pressure loss in these models relative to the water reference environment, always increases in the balloon-deformed models. Notably, the majority of the focal loss (3.5 of 6.1 dB) in the deformed M3 model is caused by phase rather than attenuation.

This is a consequence of two effects. First, the shallower the target, the greater proportion of the incident field that passes through the most heavily distorted tissue interface (see dashed red line intersections with the skin in Figure 8). This is expected to create stronger refraction of the incident field through abdominal wall. Second, the M3 case is the most laterally inhomogeneous, with the incident field passing through either stomach or liver on the way to the target.

Refraction effects are seen not only in the main lobe peak statistics, but also in the field dimensions. M3 again shows the largest increase in main lobe width, increasing from 12% to 28% wider than the ideal water beamwidth when the balloon deformation was applied. The peak sidelobes increase with the balloon for all models, but perhaps the most critical effect is in the amount of power transmitted through the field sidelobes. To assess this, the acoustic power in the focal plane of each model was calculated by spatially integrating the intensity distribution (Equation (2)) in two regions: (1) over the full field (P_TOT_) and (2) in the region excluding the main lobe (P_SL_). For all baseline tissue models, the ratio of sidelobe to total power (P_SL_/P_TOT_) is nearly uniform at 45–46%. These values rise to 50, 54 and 74% for M1–M3 and are accompanied by an increased lateral spread of several millimeters. This shift in the distribution of absorbed power away from the relatively narrow main lobe is expected to be important for moving beam therapies such as the one examined here, and even potentially beneficial in the context of inducing volumetric hyperthermia rather than ablation.

**Figure 9.**
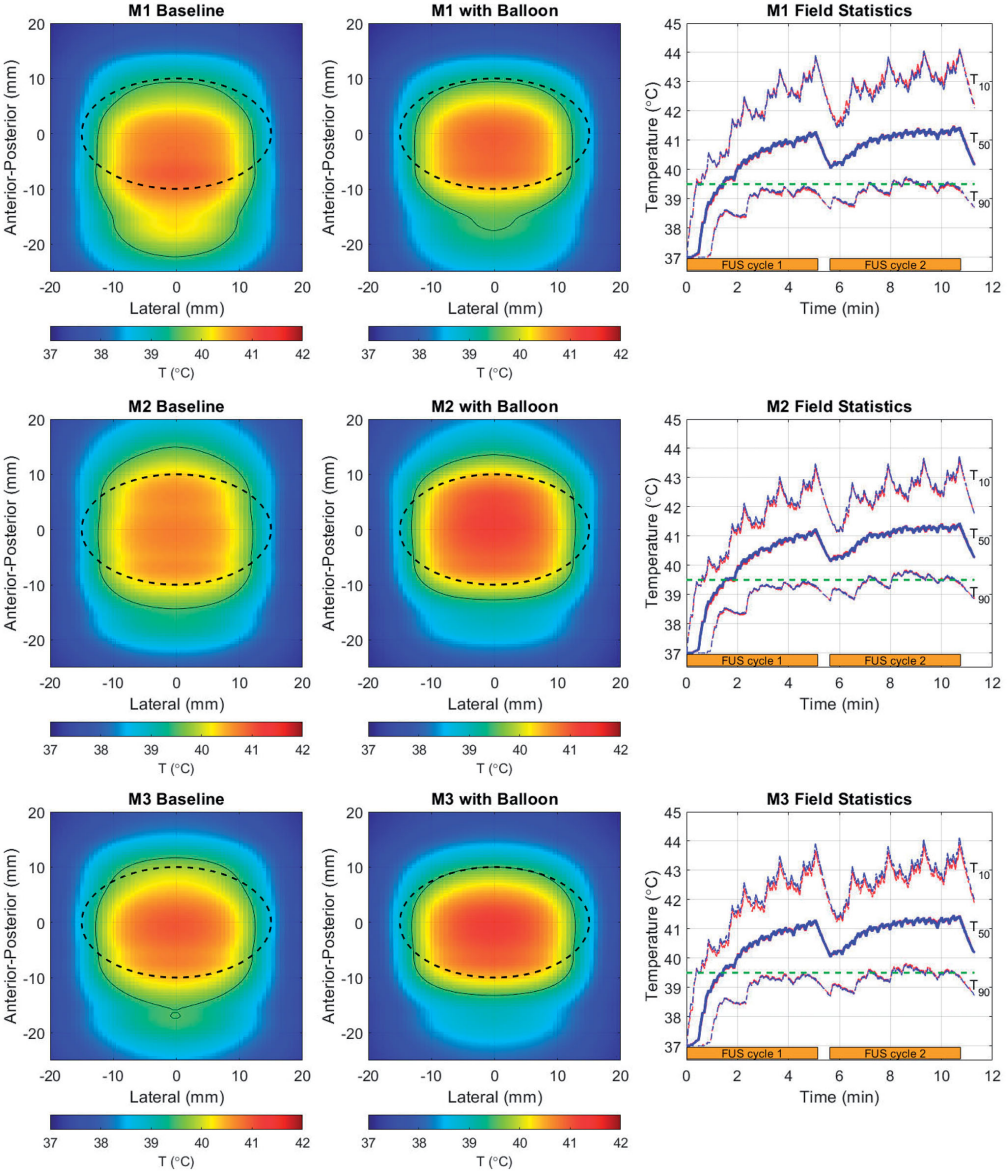
Cycle-2 time-averaged temperature maps projected in the lateral-A-P plane for the baseline (left) and balloon-deformed (center) configurations of each patient model, and histories (right) for T_50_ (thick line) and *T*_10_/*T*_90_ (thin lines) spanning two treatment cycles (orange bars). In the temperature maps, overlaid lines indicate the PDAC target (dashed black) and the 39.5 °C iso-contour (thin black). Colors in the field statistics denote the baseline (red), and balloon-deformed tissue (blue). Upper row: M1 (70 mm target depth), Middle row: M2 (66 mm target depth), Lower row: M3 (55 mm target depth).

To see the impact of abdomen geometry on tissue heating, the ellipsoidal treatment volume shown in Figure 8 with black dashed lines was given two identical cycles as described in Section 3.1. FUS power and duty cycle were set in order to achieve a 4.0 °C spatial median temperature elevation over the second treatment cycle. The results are shown in Figure 9 and compiled in Table 3.

**Table 3.** Volume heating simulation statistics for each patient model.

Model	*T*_50_ (°C)	*T*_10_ (°C)	*T*_90_ (°C)	CEM43-*T*_10_ (min)	V_ext_ (cm^3^)
M1					
Baseline	41.0	43.0	39.3	8.1	6.3
Balloon	41.0	42.9	39.3	7.5	3.5
M2					
Baseline	41.0	42.5	39.4	4.6	2.3
Balloon	41.0	42.5	39.4	5.1	0.9
M3					
Baseline	41.0	42.7	39.4	6.1	4.0
Balloon	41.0	42.8	39.4	7.4	1.6

*T*_50_: time averaged median temperature of target volume in cycle 2.

*T*_10_, *T*_90_: time averaged temperature of warmest 10% and 90% of the target volume in cycle 2.

CEM43-*T*_10_: cumulative thermal dose for cycles 1 and 2 calculated based on T_10_ time history.

*V*_ext_: volume of tissue outside the target volume ≥ 39.5 °C in cycle 2.

The cycle-2 time-averaged temperature maps shown in the left and center columns of Figure 9 exhibit some similar trends for all three patient models. The treatment volumes are generally well-covered and broadly elevated above the ThermoDox™ release threshold (thin black contour), but the heated region tends to be elongated in the A-P direction and compressed laterally. The modestly incomplete lateral heating is indicative of the elevated perfusion of healthy pancreas used in the simulations, and is readily offset by extending the width of the treatment scan lines by 3–4 mm.

Thermal field elongation is quantified in Table 3 by V_ext_, which is the volume outside the treatment target that has a time-averaged temperature increase exceeding 39.5 °C. The elongation results from a combination of factors, the first being a tendency to generate more heat proximal to the FUS source because path attenuation is lower for the shorter path length, and the HIFU system employed here does not allow for depth-dependent power adjustments within a volume scan. The second contributor is the beam refraction noted in the pressure maps. Some expansion of the heated region beyond the formally defined tumor boundary is useful for treating the tumor margins. However, the A-P elongation seen with M1 extends more than a centimeter toward the source – this appears excessive, but its impact would depend on the neighboring structures. Further control of the A-P extent of heating can be obtained by reducing the depth spacing between scan lines.

Despite the increased refraction of the pressure fields noted in the previous section when balloon-induced deformations are applied, the thermal fields do not follow the pressure trends. Instead, they tend to have shortened A-P extents outside the treatment volume. This appears to be a result of power deposition from the sidelobes, which were notably seen in Figure 8 to be broadest distal to the foci. The distal sidelobes, where $\dot{q\;}$values are very low but the volume over which they exist is high, act to thermally offset the refracted main lobe during the moving beam treatment. This is a clear distinction from static beam treatments such as ablation, where the main lobe power deposition controls the tissue heating. By way of example, examination of the M2 deformed model temperature histories indicates that the depth (A-P) centroid of heating starts approximately 2 mm below the target center. It then gradually shifts away from the FUS source throughout the first treatment cycle and stabilizes approximately 3 mm distally after approximately 6 min. These results further emphasize the relative long timescales at work in the proposed moving FUS beam hyperthermia scheme.

The heating histories in the right-hand column all look quite similar across models. As indicated by the values in Table 3, the T_90_ values are with a few tenths of a degree of the ThermoDox™ release threshold, so that efficient drug release should be possible through the vast majority of the treatment volume. Despite the *T*_10_ values peaking near 44 °C, these peaks are of short enough duration and occur far enough apart from each other that the cumulative thermal dose metrics (CEM43-*T*_10_) are all less than nine minutes.

Finally, the FUS input powers required to provide the desired level of mild hyperthermia were between 35 and 50 W at 40% duty cycle. These powers are as much as an order of magnitude lower than values reported for ablation treatments [68] and are also much lower than those used for TARDOX, which had to compensate for beam blocking by ribs in order to treat targets in the liver.

### 3.3. Respiratory motion

**Figure 10.**
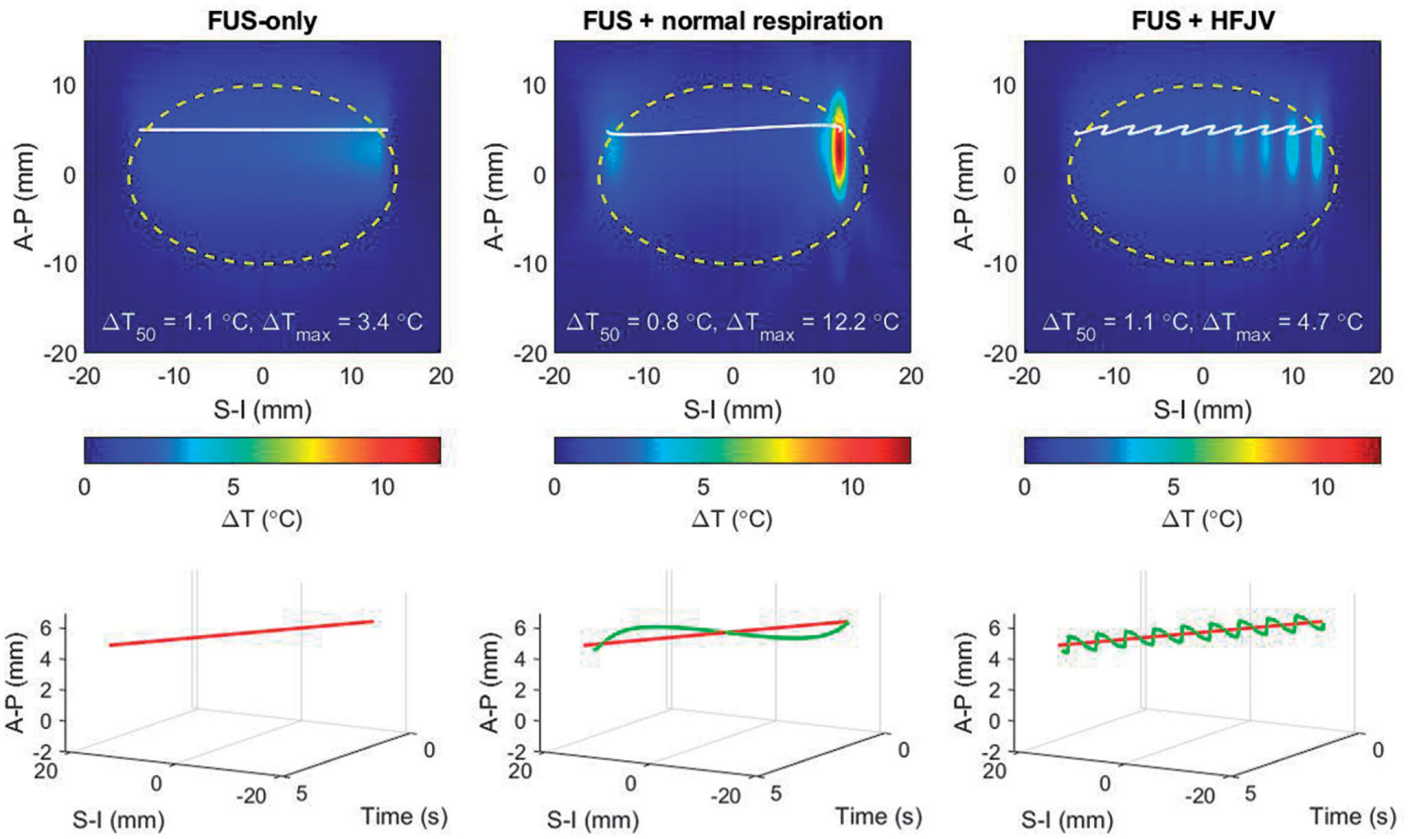
Temperature elevations for patient model M1 after a half-line moving beam exposure (4.7 s duration): left: no respiratory motion, center: normal respiratory motion, right: HFJV. In each panel, the coordinate system is referenced to the pancreas target center, the white line indicates the net trajectory of the HIFU beam, and the yellow dashed line encircles the treatment ‘slice’. The line plots below each colourmap indicate the beam trajectory time histories without (red) and with respiratory motion (green).

Simulations illustrating the effects of respiratory motion were carried out using patient model M1 with target displacements induced under normal respiration and with high frequency jet ventilation (HFJV) according to the parameters listed in Section 2.5. To begin the analysis, Figure 10 shows the temperature elevation after a single half-line beam trajectory has been completed (see Figure 3). For the simulations presented in the prior sections this represents the thermal response after 4.7 s of heat deposition spanning one-quarter of a slice of hyperthermia treatment. In Figure 10, temperature elevations within and outside the treatment slice are shown in the upper row, and the beam trajectories without (red) and with respiratory motion (blue) are shown as a function of time in the lower row.

Despite the short exposure time, the impact of respiratory motion is already clear. Looking first at the normal respiration result, when the target motion is similar in speed and direction to that of the HIFU beam, the beam ceases to traverse the target, and the stagnated exposure condition results in substantial temperature elevation relative to the HIFU-only case (12.2 vs 4.7 °C maximum temperature elevation.) This is clearly visible at the end of the line trajectory (solid white line), where the beam dwells at a nearly fixed superior-inferior position while modestly translating anterior-posteriorly. When the respiratory motion and HIFU beam trajectories are opposite, their relative motion rate increases and the traversed region is under-heated, as seen in the central S-I section of the target. The uneven heating from normal respiration produces a lower median temperature over the slice area (yellow dashed outline).

When respiratory motion is controlled by HFJV, the total displacements are smaller (1.1 vs 4.3 mm amplitude) and more frequent (2.0 vs 0.23 Hz), resulting in a relatively rapid oscillation of temperature elevation. In the example of Figure 10, the median temperature elevation in the slice volume is the same for HFJV (*T*_50_ = 1.1 C°) as it is without motion.

**Figure 11.**
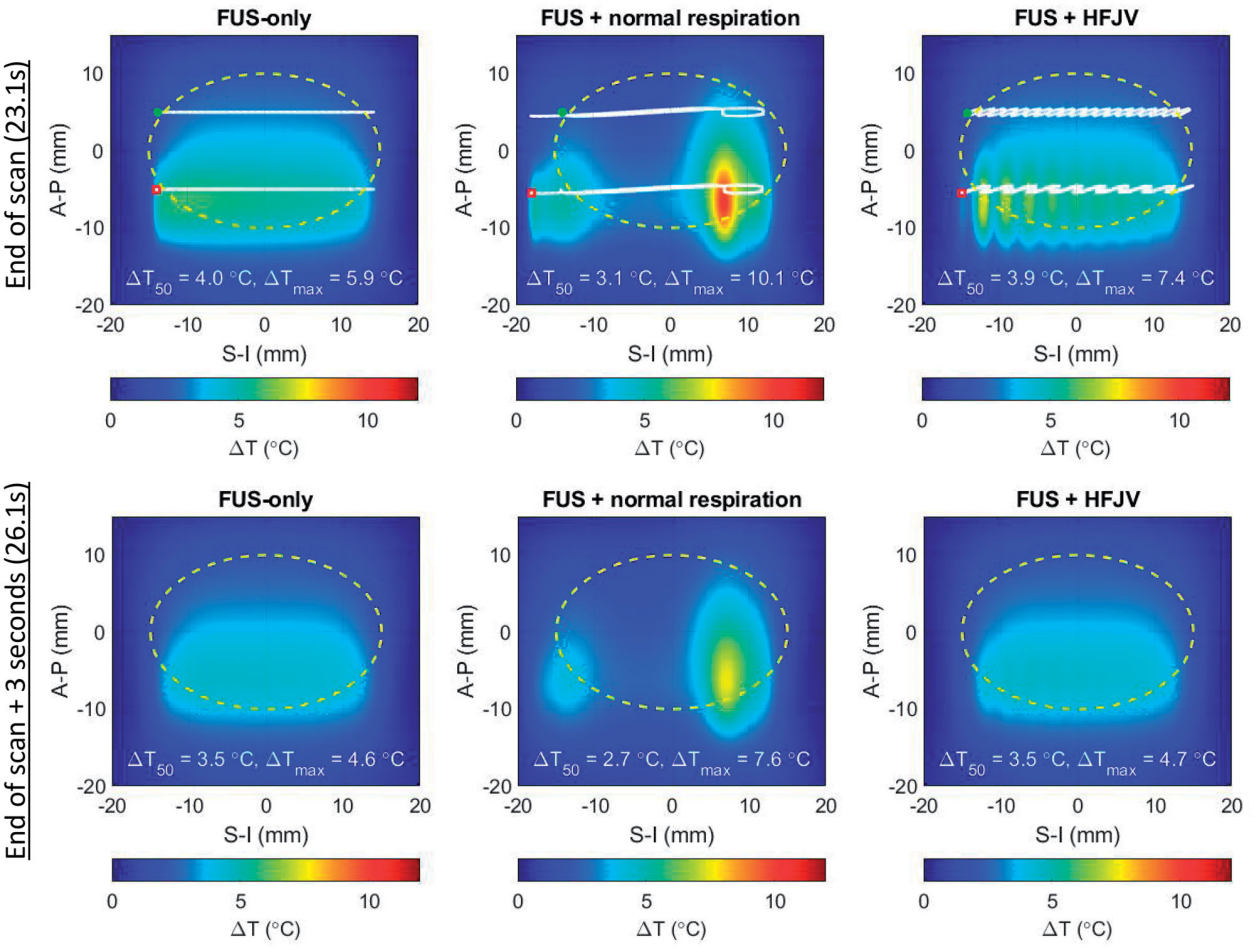
Temperature elevations for patient model M1 after a full slice moving beam scan (upper row) and 3 s after the end of the scan (lower row). left: no respiratory motion, center: normal respiratory motion, right: HFJV. In each panel, the coordinate system is referenced to the pancreas target center, the white line indicates the net trajectory of the HIFU beam, and the yellow dashed line encircles the treatment ‘slice’. The trajectory start and end points for the full slice treatment are indicated with green and red marks, respectively.

When the slice treatment has completed after 23.1 s, the highest temperatures are seen in the second scan line nominally centered at an A-P position of −5 mm (Figure 11, upper). Notably, respiration motion pushes the beam trajectory nearly 6 mm outside the lower left corner of the target region. In the time between completion of the first and second scan lines (18.4 s), the nonuniformity in the upper half of the treatment region is minimal. This demonstrates that the local over-heating is short-lived – an effect that can be more clearly seen in the lower panels of Figure 11 that show temperature elevations 3.0 s after the FUS scan ended. At that time point, the FUS-only and FUS + HFJV fields are within tenths of a degree in overall shape, median temperature, and peak temperature.

In contrast, the FUS + normal respiration field still shows strong non-uniformity, and the spatial maximum CEM_43_ was 78.2 min for the single treatment slice, compared with 0.08 and 0.16 min respectively for the FUS-only and FUS + HFJV cases.

The details of the location and magnitude of maximum temperature elevation depend on the relative motion of the programmed FUS scan and respiratory displacement history. In particular, the relative phase of respiration and the FUS scan (i.e., where in the inspiration/expiration cycle the lungs are when the FUS beam is turned on) will vary over the course of the treatment.

**Figure 12.**
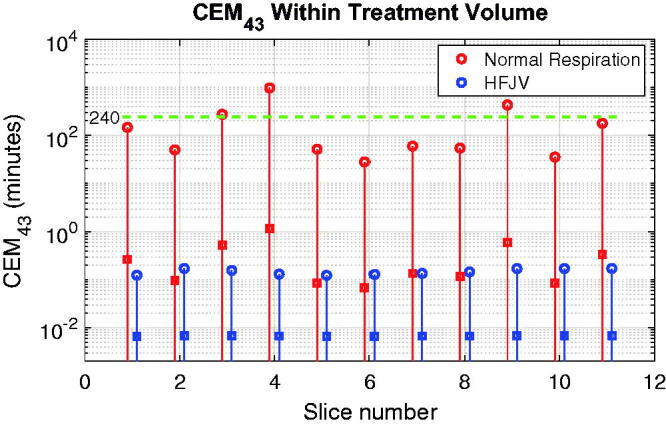
CEM43 values for each treatment slice of the standard target used in this study. Solid lines span the full range of values in each slice, and the peak and mean values are indicated by open circles and square symbols, respectively. The green dashed horizontal line denotes the critical value of 240 min.

After extending the above analysis to the 11 treatment slices covering the standard treatment volume used in this paper, the peak normal respiration CEM_43_ values in Figure 12 exceed 240 min in 3 of 11, while the HFJV values never exceed 0.2 CEM_43 min_. For those three slices in which CEM_43_ exceeded 240 min, the affected tissue volumes were 0.6, 3.2 and 12.7 mm^3^, which are 0.09, 0.47 and 1.87 times the half-intensity beam volume in water. Across all treatment slices the peak CEM_43_ values typically occurred at the depth of the more proximal scan line (6–7 mm below the target center), but the positions of the maxima in adjoining slices were separated by a mean distance of 6.8 mm, making the risk of cumulative damage in adjoining slices low in this example.

Simulations with additional breathing frequencies are shown in Supplemental Information, along with animations of single slice heating for normal respiration and HFJV. Decreasing the breathing frequency by 25% or more (as in mild sedation) markedly reduces the exposure risk, but since the respiratory motions remain large, problems with uniformity of temperature elevation and off-target heating persist.

## 4. Discussion

### 4.1. Acoustic properties

The results in Section 3.1 clearly demonstrate the critical importance of using accurate acoustic tissue properties when developing models of ultrasound-tissue interactions. In the specific context of ultrasonic hyperthermia for pancreatic cancer, the use of literature values could have resulted in underheating by 1.2 °C relative to the 41.0 °C goal, placing the median target temperature just 0.3 °C beyond the Thermodox™ release threshold. This scenario sits at the borderline between effective and ineffective drug release and would be highly sensitive to other uncertainties in the treatment. Within the scope of this study, sensitivity to attenuation values on the order of 30–40% clearly impact the volumetric heating outcomes, and it appears that the existing healthy bovine and porcine data are not adequate descriptors for human tumors. A more detailed sensitivity study will be presented in a companion paper whose focus is on the impact of the tissue properties for both mild hyperthermia and ablation [42]. Researchers seeking to evaluate candidate therapies should either seek to confirm the critical properties of the target tissues or conduct new studies to determine them in order to maximize the likelihood of successful treatment, especially for clinical applications.

The human data used in this study is the first of its kind both for normal and pathological pancreatic tissues, which further compounds the usual challenges encountered when comparing with other studies in the literature (e.g., methodological differences, measurement errors, inter- and intra-sample variability, data set size) because there are no other human data available for comparison. It is not known whether these or any postmortem data sets are suitable replacements for patient-specific *in-vivo* measurements (when feasible). Certainly, for treatment planning purposes or simulations of potential new therapeutic concepts, it is preferable to make use of the most relevant available information in terms of species and pathology.

The analyses were conducted at a single frequency (0.96 MHz) using a linear acoustic model, the predictions from which were shown to be accurate to within <10% for the ranges of pressures used in a similar prior study [20]. However, the frequency dependence of attenuation (captured by the frequency exponent) may have a significant impact if higher pressures were to be used for candidate pancreas therapies.

All heating simulations were performed under the assumption that attenuation and absorption are equal. There has been no literature assessment of this assumption in tumor tissue, but measurements of attenuation and absorption in bovine liver [69] yielded no statistical difference at frequencies of 1.1 and 3.4 MHz. There was, however, a statistical difference at 5.6 MHz where the ratio of absorption to attenuation was found to be 0.82. Similarly, this ratio was found to be 0.73 at 4 MHz in dog muscle [70]. Since the moving beam technique used for PanDox (and TARDOX) does not rely on nonlinear heating mechanisms to achieve mild hyperthermia, any high frequency differences between absorption and attenuation would not be of any consequence. Numerical methods such as those used in the present work or other ultrasound-mediated heating studies [43,71] can separately adjust the absorptive and scattering components of the total attenuation to assess the impact on heat generation, but evaluation in the context of PanDox is left to future studies.

Furthermore, the simulations in this study did not assess potential temperature or time dependence of any of the properties, and the newly collected property data was derived from fresh ex-vivo samples held at temperatures near 37 °C. The technical risks associated with these factors are thought to be low given that the application is for mild hyperthermia rather than ablation, and while some local excess heating may occur, it is extremely short-lived due to the continuously moving beam and the effects of diffusion even if perfusion was not involved. Perfusion is known to change at elevated temperatures, especially in healthy small animal tissues [72]. However, a variety of human tumors have shown minimal variation in perfusion under mild hyperthermic conditions [73]. Overall, data on human tumors remains scarce, and we are unaware of any temperature-dependent data for PDAC tumors or normal pancreas. Setting the target temperature at 41.0 °C rather than at the Thermodox™ release threshold (39.5 °C) provides some degree of robustness against potential perfusion changes that may enhance cooling, particularly at the target periphery. As for the use of *ex-vivo* tissue sample data, attenuation changes in postmortem tissues have been reported as minor [62].

As is common in bioacoustics modeling, all properties were assigned under the assumption of homogeneity within each tissue class, so local inhomogeneity in real tissues was not represented. While the impact of this simplified approach on patient-specific drug delivery is not known, the spatio-temporal smoothing provided by diffusion tends to make small scale variability less important for mild hyperthermia, at least in terms of heat deposition. Gaining a greater understanding of how extracorporeal ultrasound interacts with tissues on multiple time and spatial scales is a matter of ongoing research.

### 4.2. Abdomen deformation

This study indicates that abdomen shape is important to consider in the planning process when the shape and location of pressure maxima are of primary interest, and it is not sufficient to assume that the geometry as scanned with CT or MRI will accurately represent the conditions during the actual treatment. This is especially critical for targets in the abdomen while treating patients in an orientation that is not directly supported or maintained by the rib cage.

In this study, the water balloon deformations increased refraction of the FUS main lobe toward the FUS source while depositing a larger proportion of power in the sidelobes. However, the latter effect appeared to offset the former during hyperthermia scans. As such, the spatial distribution of temperature elevation is not necessarily coincident with or obvious from the behavior of the FUS main lobe, and the significance of this may vary depending on whether the goal is drug release, drug transport, or ablation. Specific results will be dependent on the patient as well as the balloon geometry, its inflation pressure, and the FUS device with which it is deployed. Therapies that are primarily interested in the location of pressure maxima such as ablation and histotripsy may benefit from abdomen shape studies, but these therapies typically have imaging feedback to support localization from B-mode, elastography, or MRI thermometry.

The temperature distributions in the target volume had steady state values of approximately 39.4, 41.0 and 42.8 °C for *T*_90_, *T*_50_ and *T*_10_, respectively. This spread is primarily a consequence of working with a relatively compact FUS-induced heat source (even after the tissue environment expands the sidelobes). The problem is further exacerbated when treating larger tumors as seen in the TARDOX trial, and therefore the use of an expanded beam and/or multiple beams for more efficient and uniform heating is a matter of ongoing research.

The abdomen deformations employed in this study were based on B-mode ultrasound images of patient abdomens while a water balloon was in place. This approach allowed simulation of the effects of shape-induced refraction, but with several simplifying assumptions. In particular, the deformations were applied without thickness or property changes to the constituent tissues, and the final arrangement of intra-abdominal tissue and fluid was hypothetical. The validity and limitations of this approach will be assessed as trial patient cases become available. Critically, the extent to which the stomach or duodenum can be pushed out of the FUS cone is not known *a priori*, so the present modeling approach is intended to be repeated with several variations on balloon inflation and organ displacement. The geometry that most closely resembles the B-mode display just before commencing treatment would be used for the final recommended treatment parameters. Assessment of this approach with clinical cases will be the subject of future work.

### 4.3. Respiratory motion

The spatial and temporal smoothing effects of diffusion and perfusion tend to make moving-beam hyperthermia treatments rather tolerant of small motions, which was seen in the invasive thermometry measurements during the HFJV-assisted treatments in the TARDOX trial. However, the simulations of respiratory-induced motion in the present study illustrate the risks of treatments performed under normal respiration even with the low powers used for mild hyperthermia. The modeling of normal respiratory motion and HFJV demonstrates that, even for the reduced range of motion of the pancreas when the patient is treated in the prone position, some form of respiratory control is essential to achieving uniform temperature distribution throughout the target tissue and avoiding localized regions of excessive heating. However, it does not necessarily follow that HFJV is indispensable and the only way to achieve such respiratory control. While the amplitude and frequency of HFJV-simulated displacements clearly mitigate the risk of thermal damage, other methods that do not require specialized anesthesia instrumentation and personnel are of interest [74,75]. For example, and as yet unproven, sedation could play a significant role as a compromise that bridges motion limitations with the possibility of scalable widespread adoption. In the absence of either HFJV or sedation, respiratory motion could in principle be used to assist mild hyperthermia treatments, as has been proposed for MRI-guided therapy [76,77]. The choice of HFJV in the PanDox study is related to the relative simplicity of the clinical system being used and the unavailability of gating controls even if motion monitoring and/or prediction data were available in real time.

The effects of respiratory motion were only reviewed for rectilinear scanning at one (maximum) speed available on the clinical system, but the presented framework can be readily applied for other systems and beam trajectories. Motion simulations were carried out for a limited range of simulated values for frequency, displacement in the S-I and A-P directions, and displacement shape (sinusoidal), but again, the simulations can accept any specified form of motion should such investigations be of interest. The present study did not consider non-rigid motion of the target organ or transmitted acoustic field variations due to motion-induced changes in the propagation path. Motion due to cardiovascular sources was also not considered, although the higher frequencies and lower amplitudes are less likely to have a meaningful effect on the pancreas. An exception may be if the target tumor was within a few millimeters of the aorta, but in that instance, the target would not be considered a safe candidate for ultrasound therapy. In the particular case of mild hyperthermia for targeted drug release, incidental release in the aorta would be counterproductive and increase the potential for adverse side effects.

## 5. Conclusions

‘All models are wrong…’, and in the case of clinical hyperthermia planning, a myriad of errors can arise from the necessary over-simplification of the biophysical system of interest. In the quest for a more ‘useful’ model, this work presents refinements to our prior clinically validated treatment planning model for mild hyperthermia to include three realistic factors: improved knowledge of target tissue properties, more accurate representations of body morphology as treated, and the inclusion of respiratory-induced motion. This introductory study indicated that respiratory motion was of highest potential significance, followed by pancreas properties and abdomen shape.

Human pancreas ultrasonic properties collected in a companion study showed a substantially lower attenuation than was previously available in the literature. Carrying this through to volumetric hyperthermia simulations showed that reliance on literature attenuation values could result in borderline-sufficient hyperthermia for Thermodox™ release, with partial undertreatment and little margin for tolerance of other clinical uncertainties. This could be particularly detrimental to therapy concepts that do not employ real time thermometry.

When a water balloon is installed to compress the stomach and/or push air-filled organs out of the FUS field, the resulting simulated abdomen deformations cause substantial changes to the pressure field, most notably through refraction toward the FUS source and attenuation of the main lobe. However, these changes are offset during moving beam hyperthermia by the action of the sidelobes over several minutes. As such, the impact of abdomen curvature on mild hyperthermia as implemented in this study was small. If the size and location of the pressure field was important, as in the case of enhancing drug transport, then the curvature modeling would still be of value.

Simulations of respiratory-induced motion showed substantially elevated risk of tissue damage and degraded temperature field uniformity, both of which would be detrimental to treatment concepts relying on mild hyperthermia. Motion amplitudes and their adverse effects were minimized through the use of HFJV, yielding thermal fields that were nearly identical to those where there was no respiratory motion.

Each of the above components in this study is a useful step toward realism in FUS treatment planning for abdominal targets, and in combination can help inform clinicians of risks during the planning stage and allow for treatments to proceed with potential for both improved safety and efficacy. There is still much work ahead, and it is hoped that this paper serves as a springboard for further development toward model-facilitated treatments that require lower cost facilities without sacrificing overall treatment quality and outcomes.
